# Ab Initio Molecular Dynamics Study of Phospho-Amino Acid-Based Ionic Liquids: Formation of Zwitterionic Anions in the Presence of Acidic Side Chains

**DOI:** 10.1021/acs.jpcb.9b09703

**Published:** 2020-02-10

**Authors:** Henry Adenusi, Andrea Le Donne, Francesco Porcelli, Enrico Bodo

**Affiliations:** Chemistry Department, University of Rome “La Sapienza”, Piazzale Aldo Moro 5, 00185 Rome Italy

## Abstract

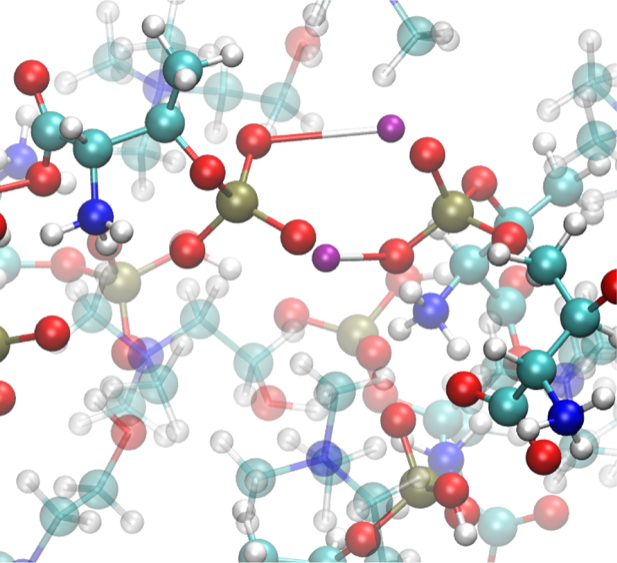

We present a computational analysis of the complex proton-transfer processes in two protic ionic liquids based on phosphorylated amino acid anions. The structure and the short time dynamics have been analyzed via ab initio and semi-empirical molecular dynamics. Given the presence of mobile protons on the side chain, such ionic liquids may represent a viable prototype of highly conductive ionic mediums. The results of our simulations are not entirely satisfactory in this respect. Our results indicate that conduction in these liquids may be limited due to a quick quenching of the proton-transfer processes. In particular, we have found that, while proton migration does occur on very short timescales, the amino groups act as proton scavengers preventing an efficient proton migration. Despite their limits as conductive mediums, we show that these ionic liquids possess an unconventional microscopic structure, where the anionic component is made by amino acid anions that the aforementioned proton transfer has transformed into zwitterionic isomers. This unusual chemical structure is relevant because of the recent use of amino acid-based ionic liquids, such as CO_2_ absorbent.

## 1 Introduction

One of the key features of protic ionic liquids (PILs) is the presence of mobile protons that can act as light and fast charge carriers. Typically, a PIL is made by a deprotonated Brønsted acid (the anions) and a protonated Brønsted base (the cations). A very simple example is ethyl ammonium nitrate,^1^ where the nitric acid has formally lost its proton that resides on the ammonium ion.

The possible activity of the mobile protons in PILs not only has important effects on the fluid structure and on its frictional properties,^2^ but has also spawned an intense research activity because such particles might act as charge carriers^3^ (in addition to the ions themselves), hence prompting the use of PILs as highly conductive materials in electrochemistry.^4−8^ In addition to the electrochemical implications, PILs are gaining further attention because they display an array of complex intermolecular interactions as well as a peculiar chemical activity, due to their complex hydrogen-bonding features.^9−12^

Recently,^13^ it has also been shown that ionic liquids can be used for CO_2_ capture and therefore serve as greenhouse gas scavengers. It turned out that amino acid-based PILs seem to be particularly efficient in absorbing CO_2_ because they capture it by chemisorption through the formation of carbamates.^14,15^ It therefore follows that the study of the amino acid-based ionic liquid morphology at the molecular level is crucial for understanding and optimizing CO_2_ absorption.

Despite the apparent simplicity of their synthesis, which consists of an acid–base reaction, and of their widespread use as solvents for electrochemistry, the mechanisms at the origin of the conductivity and frictional properties of PILs are still poorly understood. For example, it has recently been shown^16^ that PILs in which proton transfer is incomplete (i.e., they contain a sizable amount of neutral molecular species) show a significant proton conductivity. In certain PILs, proton transfer can be enhanced by a Grotthuss-like mechanism^17^ when a neutral molecule, such as imidazole, is added to the liquid. A similar mechanism has been detected in PILs with partially neutralized diamines.^18^ Further insights into the complexity of certain PILs has been obtained by us;^19−21^ we have shown that proton transfer in fully ionized liquids can be mediated by the clustering of like-charge ions (specifically amino acid anions).

As we mentioned above, PILs are generally produced using equimolar amounts of Brönsted acid and base. Proton transfer from the acid to the base leads to ionization and to the formation of proton donor/acceptor sites that spawn a hydrogen-bonding network. The degree of ionization in a PIL is dependent on the Δp*K*_a_ between the acid and the conjugate acid of the base. It is understood that a large difference in the p*K*_a_’s of the neutral reagents (>6) ensures that the resulting liquid is fully ionized.^22^ In this case, the proton is strongly bound to the base and further proton transfer mechanisms consist only of sporadic events that do not contribute to the bulk conductivity, which remains due exclusively to the ion drift in the bulk fluid (sub-ionic regime).^23−25^ When the difference in p*K*_a_ is small, a certain fraction of the molecular constituents remains neutral, hence causing a partial loss of the IL properties. In this case, different behaviors of the fluid can be noticed: Sometimes,^26^ the liquid is a homogeneous mixture of ionic and polar phase, and the loosely bound protons might give rise to an unexpectedly high conductivity; sometimes,^27,28^ the ionic and polar parts separate, and the evaporation of the neutral component occurs, which is certainly not desirable for practical applications.

**Figure 1:**
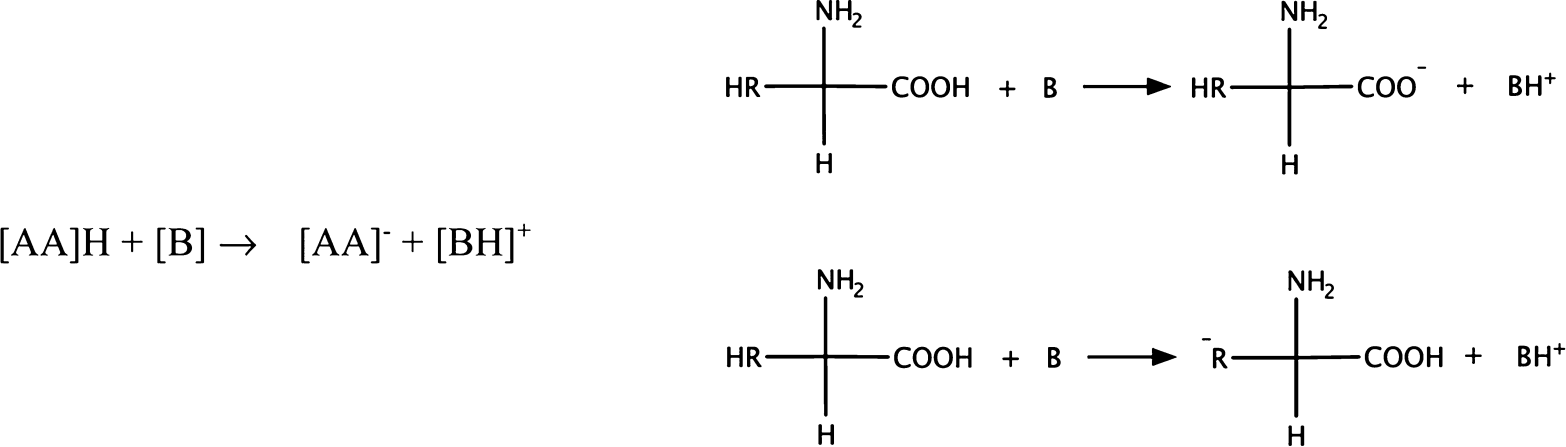
Ionic liquid formation through a primary acid–base proton transfer. BH^+^ in our case is the cholinium cation.

In the past years, we showed that it is possible to overcome this latter problem by identifying a subset of PILs, which possesses mobile protons, but in which the occurrence of a neutral moiety is only a transient event, which should not induce dramatic effects in the bulk structure. Such ionic liquids are made by cholinium cations (hereafter [Ch]^+^) coupled to amino acid (AA) anions (general formula HR–CH(NH_2_)–COOH) that have a protic function on the side chain (−HR) with a different acidity with respect to the primary carboxylic function. In these compounds, as shown in Figure 1, the primary deprotonation of either the carboxyl group or the −RH group gives rise to the AA anion and the resulting [Ch]^+^.

**Figure 2:**
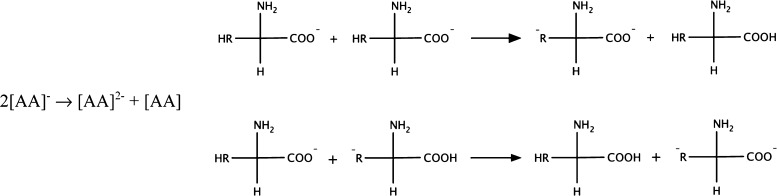
Two possible secondary proton-transfer processes taking place between anions.

**Figure 3:**
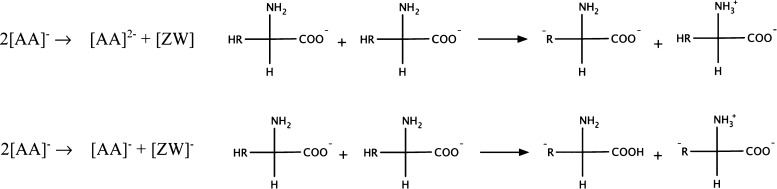
Possible examples of zwitterion and zwitterionic anion formation due to secondary proton-transfer processes.

The remaining proton that still resides on the [AA]^−^ anion can undergo secondary proton transfer equilibria, such as those reported in Figure 2, where a second anion acts as a proton acceptor. We underline that the formation of the dianion [AA]^2–^ and of a neutral [AA] is only a transient event due to the tendency of the liquid to remain ionized. While both −RH and −COOH can participate in the proton-exchange equilibria, there is also a third actor that plays a role: the anions can act as proton acceptors via their amino (−NH_2_) groups. The processes where the −NH_2_ group acts as a proton acceptor can give rise to both a neutral ([ZW]) and a negatively charged ([ZW]^−^) zwitterionic form of the AA anion. Two possible mechanisms, among others, are reported in Figure 3.

In the past few years,^29^ we have studied the occurrence of such processes in different PILs with different AA anions. In particular, we have recently analyzed the cases where the AA anions had either an additional carboxylic function or a thiolic one on their side chains. It turned out, as expected by p*K*_a_ values, that the −SH group is a relatively poor proton donor and that the secondary proton transfers are only sporadic events, very likely unable to sustain any enhanced conductivity. This finding is indirectly confirmed by the fact that the measured conductivity of a cysteine-based PIL does not deviate significantly from that of other PILs with non-protic AA anions.^30^ The AA anions with an additional −COOH group on the side chain (such as aspartic and glutamic acid), instead, turned out to be very efficient proton donors, at least in our simulations. Unfortunately, even though the transfer from −COOH does provide a mechanism of proton migration inside the liquid, its extent is limited by the high stability of the ensuing zwitterionic structures (see Figure 3). In other words, the amino group on the AA anions acts as a scavenger of protons and eventually quenches most of the migration processes in the fluid.

Despite the lack of computational evidence for a high conductivity, the recent analysis of these PILs has allowed us to highlight an important feature that is the result of an often overlooked complexity of these systems: in order for proton transfer to occur in a completely ionized medium with a chemically inactive cation, anions have to cluster together. This means that anions must be able to overcome the repulsive coulombic interaction thanks to a combination of dielectric screening, charge delocalization, and the presence of H-bonds. We have already presented computational evidence that this unusual interaction between like-charge ions can have a sizable effect on bulk properties.^21,31^ The aggregation of ions in concentrated electrolyte solutions and ionic liquids has been the subject of an intense activity of research in electrochemistry and in physical chemistry. It is well known that the molecular ions of an electrolyte in solution tend to aggregate when its concentration increases.^32^ This behavior has been shown to be induced by the solvent and to be dependent on solvent chemical properties, such as hardness.^33^ At a difference with this situation, neat ILs contain no solvent and can be considered as pure electrolytes. The aggregation of like-charge ions in ILs has been shown to occur, nevertheless, especially when large molecular structures are involved:^34−37^ in this case, however, the driving force for aggregation is provided by cooperative H-bonding effects. In our case, the stabilization of purely anionic aggregates occurs for the same reason.

**Figure 4:**
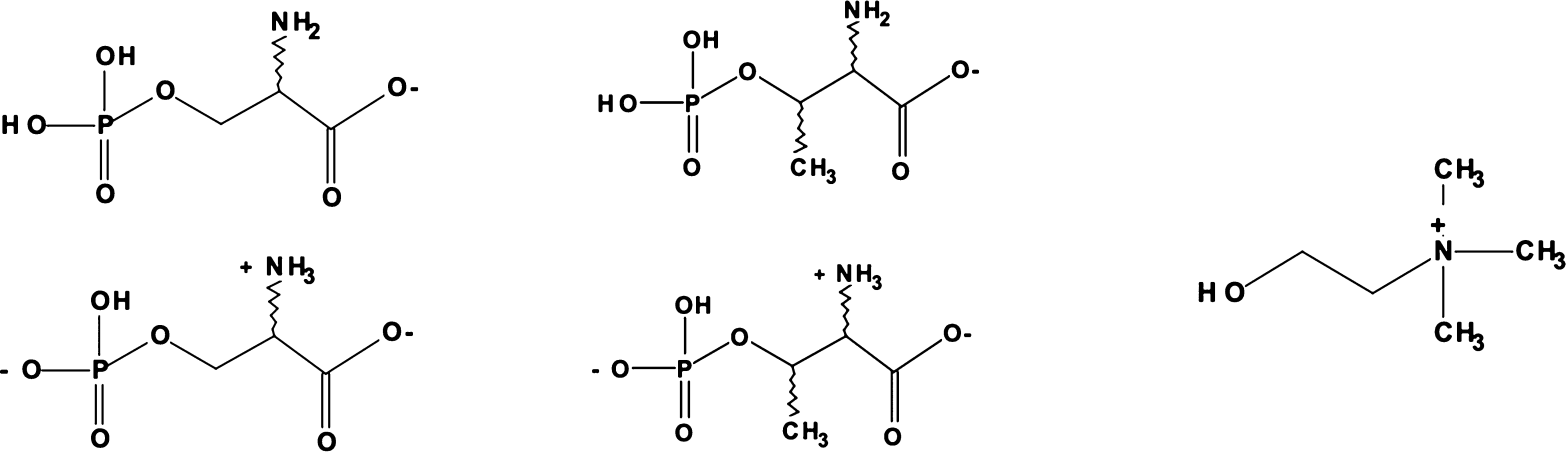
Three molecular species that are the basis of the material analyzed ion in this work. Left: [Pse]^−^ anion in the −COO^–^ isomeric form and in its zwitterionic anionic form. Center: same for [Pth]^−^. Right: [Ch]^+^ cation.

In this work, we have decided to expand the range of compounds we have examined so far and explore, by means of molecular dynamics (MD) techniques, the proton-transfer dynamics in PILs, where the AA anions contain a phosphate group on the side chain. Two compounds have been studied: phospho-serine (hereafter [Pse]^−^) and phospho-threonine (hereafter [Pth]^−^), both coupled to [Ch]^+^. The three molecular species are reported in Figure 4. As we shall see, the nature of the phosphate side chains makes the behavior of these fluids different with respect to those with a carboxylic one. In particular, the quenching of proton migration due to the amino group is less effective because the phosphate group has more than one proton that can act as a charge carrier in the fluid.

## 2 Methods

It has been long recognized^38^ that hydrogen bonds interactions and geometries are hard to model correctly, especially when using force field-based methods that often employ fixed atomic charges. At the same time, gas-phase isolated pair computations are difficult to converge to the correct ionic (charge-separated) structure because of the natural tendency for isolated molecules to mutually neutralize.^10^ Implicit solvent models provide a partial solution to this problem.^11^ In order to perform MD, either a polarizable force field has to be employed, or electronic density has to be taken into account to calculate fluctuating atomic charges. It has been shown that methods such as ab initio MD (AIMD)^39^ or semi-empirical MD, such as density functional tight binding (DFTB),^12^ are able to properly describe hydrogen-bonding features with a very good accuracy level.

In addition, the modeling of the bulk fluid, for these specific systems, requires the use of computational techniques that do not imply a fixed topology of the chemical structures or fixed atomic charges. These fluids are characterized by various bond-breaking processes, where the topology of the various X–H bonds and the atomic charges (hence the molecular polarity) is continuously changing. Therefore, the only way to reliably approach the study of proton-transfer processes in ionic liquids is represented by methods that are based on the evaluation of the “atomic” forces from an approximate solution of the electronic Schrodinger equation (AIMD). Although following the above argument, AIMD certainly provides a robust choice for this study, it suffers from two main pitfalls: (i) the resulting simulations are very demanding so that their time span is limited to a few tens of picoseconds, and (ii) the size of the systems that can be treated using this approach is limited. Due to these intrinsic limitations, our computations cannot provide a reliable thermodynamic model of the fluid at equilibrium (which would require us to simulate times of the order of nanoseconds) but, nevertheless, can provide a decent description of its local structural features and of the basic molecular processes that drive the proton diffusion. One way to improve the sampling time in our simulations is by using a less-expensive way to solve for the electronic energy and gradient. We have therefore also attempted to describe the two fluids using the semi-empirical method based on DFTB. The quality of this approximation has been assessed using its outcomes vis-a-vis those obtained using ab initio DFT.

Preliminary, ab initio calculations have been carried out on the isolated AA anions in their various tautomeric forms ([ZW]^−^ and [AA]^−^). Given that some of these tautomers have charge separations (zwitterions) and are highly unstable in vacuum, we have included an environmental dielectric screening. All the ab initio calculations have been therefore performed in a continuum solvent model (PCM). Since the dielectric permittivity of these compounds has yet to be stated in the literature, we have used the acetonitrile PCM parameters as a realistic model solvent as its dielectric constant (ε = 35.7) matches the typical value of other PILs.^40,41^ For each tautomeric structure, we have performed a fully unconstrained optimization and evaluated the harmonic frequencies using B3LYP-D3^42^ with the 6-311+G(d,p) basis set. This combination is suitable for such a study since it provides results that are comparable to the more accurate D3-B2PLYP functional, as was verified in one of our recent studies.^31^ The Gaussian16^43^ package was used for the ab initio calculations.

The bulk simulations of the [Ch][Pse] and [Ch][Pth] systems have been performed with Car-Parinello MD and the CPMD code^44^ using a cell with a side length of around 21 Å filled with 24 ionic pairs. CPMD has been performed employing Troullier–Martins pseudopotentials and the BLYP functional. The choice of the functional is due to the better performance of BLYP^31^ with respect to PBE in reproducing high-quality ab initio data for proton-transfer barriers. The production time is 33 ps for [Ch][Pse] and 23 ps for [Ch][Pth] at 300 K in the *NVT* ensemble with the temperature held constant by means of a Nosè–Hoover thermostat. In order to prepare the initial configurations for the AIMD simulations, we used classical molecular mechanics and the MM3 force field. The classical systems have been simulated using the *NPT* ensemble in order to get appropriately packed configurations with densities of 1.28 and 1.29 gr/cc for Pse and Pth, respectively. Initially all acidic protons were placed on the phosphate groups and the carboxylate ones were deprotonated.

For the semi-empirical method, we have adopted the most recent extension to the DFTB method, DFTB3,^45−47^ which includes the extensions of DFTB energy up to the third order, which is crucial for an accurate study of hydrogen bonding.^48^ The DFTB calculations were carried out by the DFTB+ program,^49^ using the mio-0-1 set^50^ and including dispersion forces by the Slater–Kirkwood polarizable atom model. Initial configurations have been created in the same way as for the AIMD simulations above. The DFTB simulations have been carried out in the *NVT* ensemble on periodic cells made by 8 ionic couples with density set to 1.25 gr/cc. The temperature was held constant using a Berendsen thermostat. The production times have been set to about 300 ps for each cell. In order to validate the semi-empirical approach, proton affinities (PA) calculated ab initio at the D3-B3LYP/6-311+G** level have been compared with those obtained using DFTB. The numerical data that provide a validation of the DFTB method are reported in Section S1 of the Supporting Information.

## 3 Ab Initio Reference Data

We have computed the relative energy of the possible tautomeric forms of the AA anion using the well-tested D3-B3LYP/6-311+G(d,p) method. The results are summarized in Table 1 where we report the energy differences between the various deprotonated states of the AA and the anionic structure (indicated as COO^–^) arbitrarily chosen to be the reference.

**Table 1:** Relative Energies in kcal/mol at B3LYP-D3/6-311+G(d,p) of the Tautomeric Forms of the Two AA Anions^a^

	Pse		Pth	
	vacuo	PCM	vacuo	PCM
COO^–^	0	0	0	0
PO_4_H^–^	0.5 (0.4)	–0.2 (0.2)	–2.7 (−2.6)	–0.5 (0.1)
ZW	5.6 (6.0)	–3.1 (−1.2)	4.7 (5.2)	–3.0 (−1.1)

a The tautomer labeled COO^–^ is a deprotonated AA that has lost the carboxylate proton, the one labeled PO_4_H^–^ has lost the proton on the phosphate, and the tautomer labeled ZW is an anionic zwitterion with the COO^–^/PO_4_H^–^/NH_3_^+^ combination. The numbers in parentheses include zero-point energy corrections at the harmonic level.

As expected, in vacuo computations tend to stabilize the two anionic forms of the AA anion with respect to the zwitterionic one. In particular, deprotonation at −PO_4_H_2_ is preferred for [Pth]^−^, while deprotonation at–COOH is preferred for [Pse]^−^. In both cases, the zwitterionic anion is, by far, the most unstable isomer with about 4–5 kcal/mol difference. An opposite situation is detected when performing the same calculations in a dielectric medium modeled through the PCM computational scheme. In both cases, a stabilization of about 3 kcal/mol is seen to occur for the zwitterionic anion with respect to the −COO^–^ form.

This result clearly points to the amino group to be the preferred residing group for the excess proton. The ab initio computation is not enough, though, to obtain a reliable answer. Despite the use of a continuum solvation model, these computations suffer from a lack of realism under many aspects: PCM is not an “exact” way of representing a solvent, and solvent–solute H-bonds are not taken into consideration. For these reasons, we have performed several MD simulations to provide a more substantial theoretical basis to our conclusions. These MD simulations are the main subject of this work.

## 4 Ab Initio Molecular Dynamics

**Figure 5:**
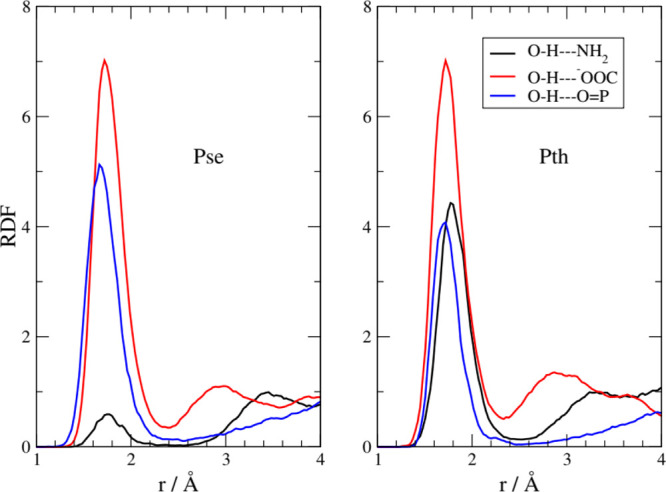
Intermolecular (cation–anion) RDFs between the hydroxyl hydrogen of cations (−OH) and different groups of the anions.

Ionic liquids are held together by the strong electrostatic interaction between anions and cations. In our case, however, both charged moieties are quite bulky, and the charges are either delocalized (the carboxylate groups) or strongly screened (the positively charged nitrogen in cholinium is sterically surrounded by methyl groups). For these reasons, the electrostatic interactions are weakened, and one of the main contributions to the anion–cation interaction comes from hydrogen bonding (HB). The HBs can be formed by the donor oxygen on the cholinium hydroxyl and the various donors and acceptors in the anion: the carboxylate, the phosphate, and the amino group. The presence of these interactions is made clear by the results reported in Figure 5 where we show the radial distribution functions (RDF) between the hydroxyl hydrogen (cation) with the mentioned acceptor groups in the anion. As expected, the strongest interaction, in both the [Ch][Pse] and [Ch][Pth] liquids, is due to the carboxylate group (red line). In both systems, however, the phosphate is also able to coordinate an HB with the cation (blue line). The average H–O distance for the carboxylate is between 1.7 and 1.8 A and that for the phosphate, between 1.6 and 1.7 A. A third interaction that appears to be active is due to an H-bond between the cationic −OH and the −NH_2_ group (black line). This interaction is less pronounced in the [Ch][Pse] liquid. This is probably due to a minor conformational mobility of [Pth]^−^ with respect to [Pse]^−^ due to the O–C–C–N torsional barrier. In both liquids, we have not found an appreciable interaction between the −OH of cholinium and the −OH of the phosphate group, as expected.

As we have pointed out above, one of the most striking features of AA-based PILs is the fact that anions do bind together (like-charge clustering). The high electric screening, the charge delocalization, and the molecular size, all contribute to this effect, which is otherwise very unlikely to occur in other salts. Once anions cluster together, a proton transfer can occur, as shown in the schemes of Figures 2 and 3.

**Figure 6:**
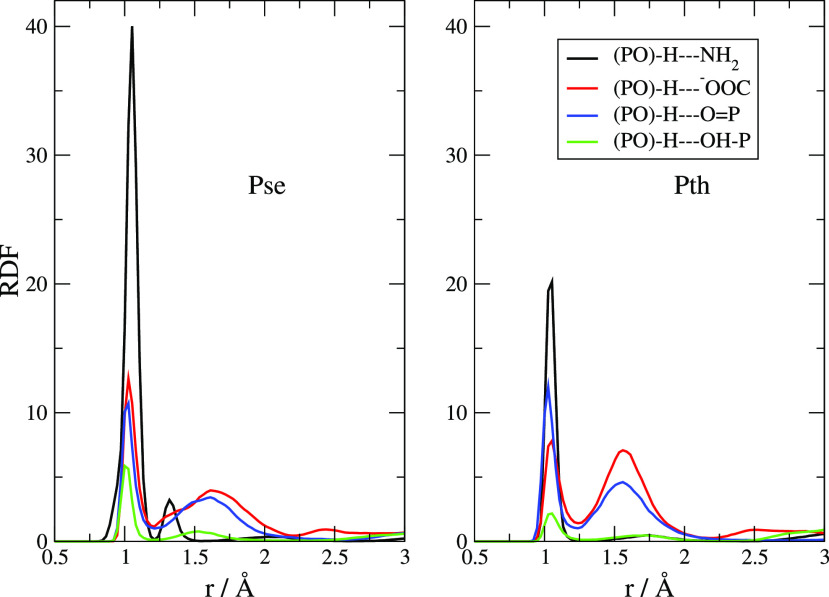
Intermolecular (anion–anion) RDFs for various protic sites toward the phosphate group.

In Figure 6 we report the inter-anionic RDFs between various acceptor sites and the phosphate group. The distances reported are those between the proton on the phosphate and the acceptors located on another anion. A variety of H-bonds have been formed between the anions, and these distances involved show that the shared protons are quite bound to the acceptor sites. The main peaks are located around 0.9–1.2 Å, and one can compare these values with those typical of the cation–anion ones that are around 1.5–1.9 Å (Figure 5). The prevalent one is due to a phosphate–amino interaction (black line), and only slightly less important in occurrence is the interaction with the carboxylate (red line). Other interactions are also at play, and we have detected them between the phosphate groups (green and blue line). While the proton-acceptor distances in both ionic liquids point to very strong H-bonding features, with the proton localized very close to the acceptor, in [Pse]^−^ there is a clearly visible shoulder on PO_4_H_2_/NH_2_ RDF at short distances (black line, below 1 Å) that can be interpreted as the result of proton transfers from PO_4_H_2_ to NH_2_.

**Figure 7:**
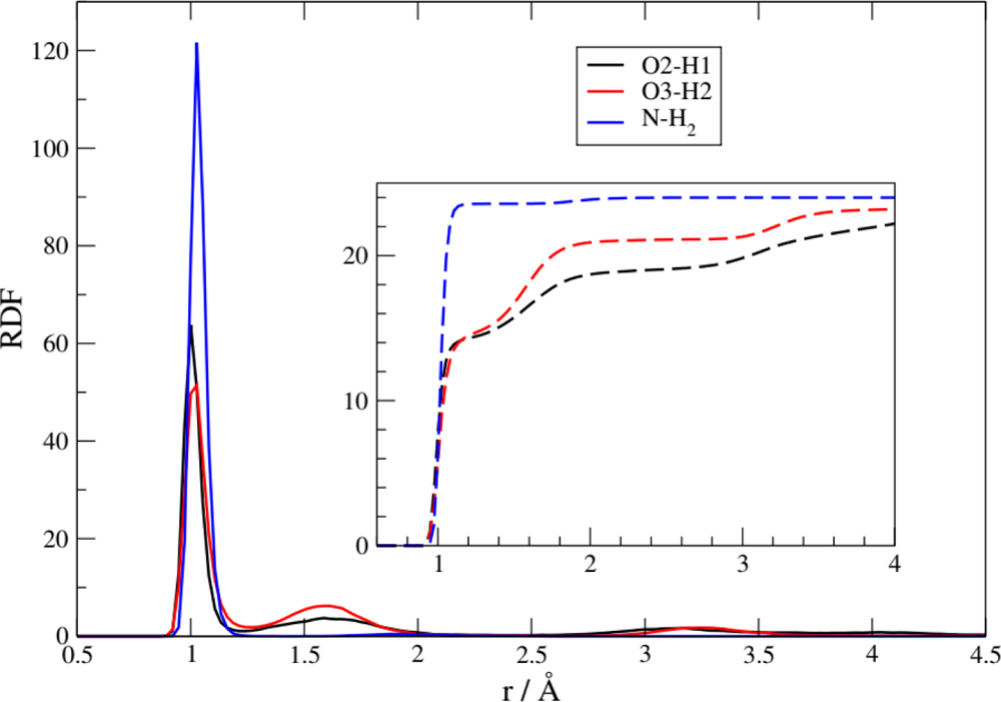
Intramolecular RDFs of [Pse]^−^ for specific bonds involving H atoms. The inset shows the running integral of the three RDFs, that is, the average number of protons at a given distance.

Occurrences of proton transfer can be easily seen if we look at intramolecular RDFs, where we pick the distances of the mobile protons from the atoms of the anions. In Figure 7, we report the results we have obtained for the [Ch][Pse] liquid. The main panel shows the intramolecular RDFs for the O–H bonds of the phosphate (red and black lines for both oxygen atoms) and that of the N–H (blue line). The inset shows the respective coordination numbers (i.e., the running volumetric integral of the RDFs) computed as
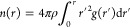


Initially, at time zero, all 48 protons are localized on the phosphate groups on both the oxygen atoms (O2 and O3). During the simulation, 20 of them separate from the phosphate and migrate inside the cell, as proven by the two RDF maxima at 1.7 and 3.2 Å. As one can grasp from the inset in Figure 7, at least 10 protons have migrated more than 3.0 Å into the fluid.

In Figure 7, we have also reported, for comparison, the N–H distances of the amino groups that do now show evidence of any proton loss (the RDF is zero beyond 1.2 Å), as expected.

**Figure 8:**
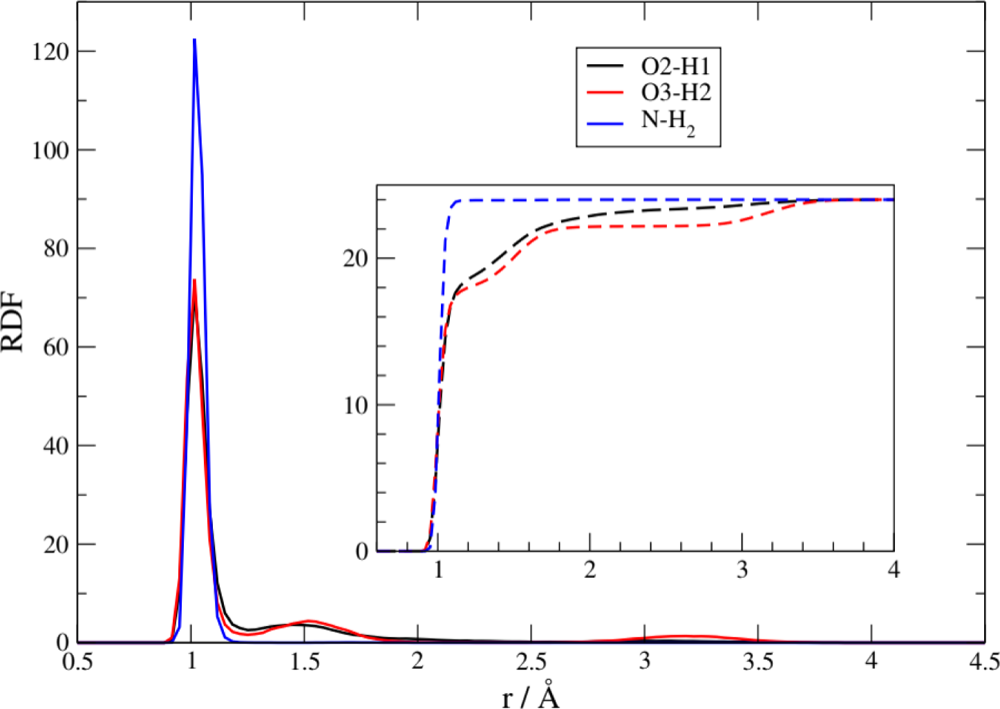
Intramolecular RDFs of [Pth]^−^.

A similar result has been obtained for [Ch][Pth] and is reported in Figure 8. Only 12 protons leave the phosphate group, which is roughly half the number of migrations detected for [Pse]^−^. While this may point to a substantial difference between the two amino acid anions, it is difficult for us to provide evidence for a real kinetic or thermodynamic effect, given the very limited time span of our simulations. In other words, the different behaviors of the two AA can be simply a consequence of a memory of the initial configurations.

Despite the above limitations, we are able to answer the crucial question: Where do these protons migrate? The short answer is that they tend to migrate mostly on the amino group. However, different from other AAs, such as glutamate and aspartate,^21^ phosphate is an acid strong enough to protonate the carboxylate groups also. As examples of this multifaceted behavior, in the following, we highlight some of the occurrences that we have spotted in our simulations.

**Figure 9:**
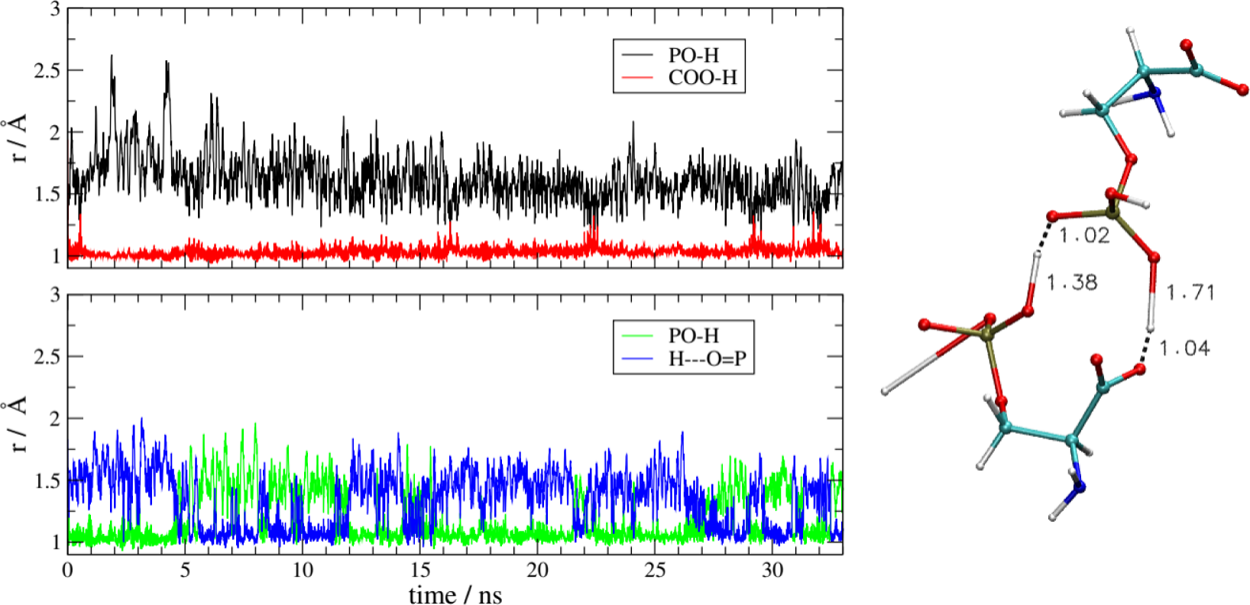
[Ch][Pse]. Left: O–H distances as a function of simulation time in a cluster made by three anions. Upper panel: migration of a proton from a phosphate to the carboxylate of another anion. Lower panel: exchange of protons between two phosphate groups (see text for details). Right: snapshot of the two anions which are taking part in the double proton exchange isolated from one of the frames of the MD simulation.

In Figure 9 we report an example of two different proton-transfer processes. Both transfers take place within a cluster of three anions formed in the bulk due to H-bond interactions. For clarity, on the right side of Figure 9, we report a snapshot of two of the three anions as extracted from the final frame of the simulation. The upper panel reports the distances of the proton from the phosphate (O–H distance, in black) and from the carboxylate (O–H distance, in red). Initially, the proton is on the phosphate, but during the short equilibration phase of the simulation (at *t* < 0, not visible on the graph), the proton has moved onto the carboxylate (the red distance is shorter than the black one). This transfer is not reversible, at least in the relative short time of our simulation. The final arrangement of the two anions, on the right, shows this proton to reside at 1.7 Å from the phosphate and 1 Å from the carboxylate. Within the same anionic cluster, another proton transfer has taken place and the distances pertaining to it are shown in the lower panel. This time, the proton is shared between two phosphate groups (blue and green distances). The motion between two phosphates is reversible, and the proton literally “jumps” between the two oxygen atoms. This behavior is consistent with the existence of a strong, charge-assisted H-bond between the two oxygen atoms of the phosphate groups. The interesting fact is that, for this strong H-bond to occur, one of the phosphates (the one at the bottom in the structure on the right of Figure 9) has lost the other proton toward another anion, as can be seen by the rather elongated O–H bond visible at the bottom-left side of the molecular structure. We see that, as soon as the AA anion acquires a positive charge on its carboxylate, it tends to maintain a global negative charge by losing another proton from the side chain. In other words, the liquid, at the molecular level, tends to remain ionized and to counterbalance the proton movements.

**Figure 10:**
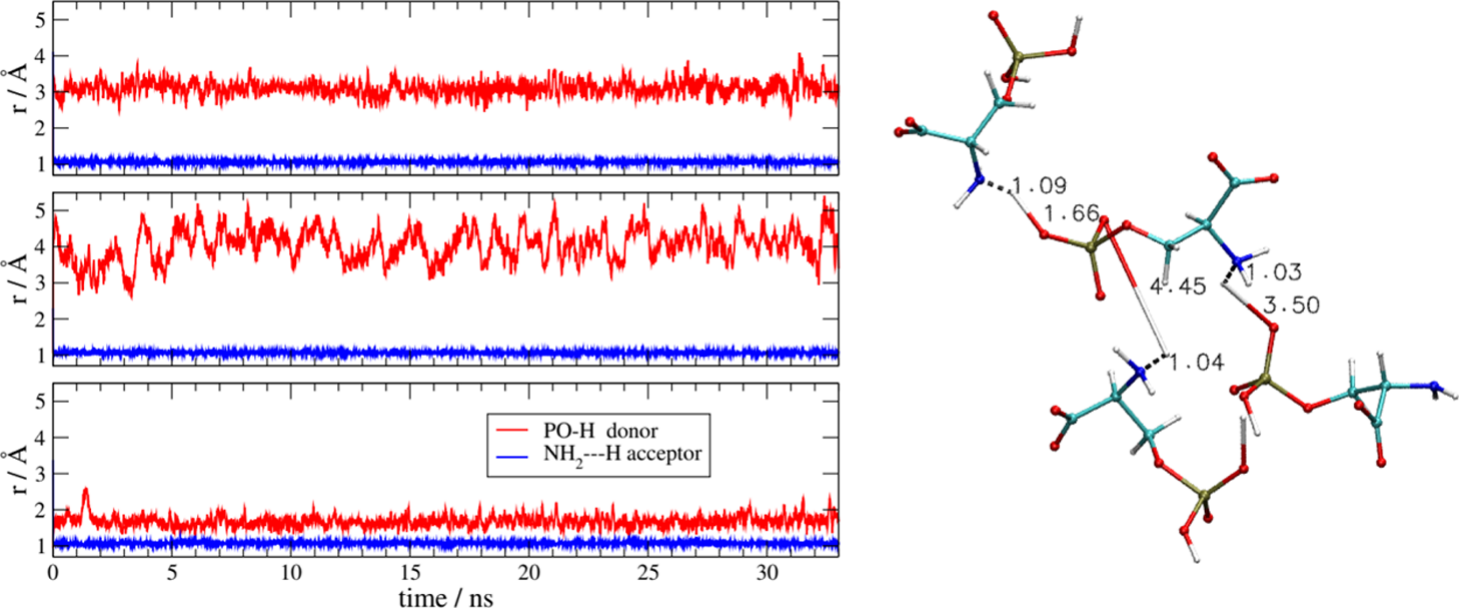
[Ch][Pse]. Left: PO–H and NH_2_–H distances as a function of simulation time in a cluster made by four anions. Right: snapshot of the four anions involved in the proton exchange, isolated from the final frame of the MD simulation.

In Figure 10 we show another mechanism of proton transfer. The proton exchange takes place between the phosphate groups and the amino group. The structure involved is a rather extended cluster made by four AA anions, which is presented on the right of Figure 10. Initially, all carboxylates are deprotonated; each phosphate group has two protons and the amino groups are neutral. After equilibration (at *t* < 0, not visible on the scale of Figure 10), three phosphates lose their protons toward the amino groups: the N–H distances (blue lines) are small and the O–H ones are large (red lines). It is interesting to note that one of the three protons described by the distances reported in Figure 10 (the one in the lower panel that corresponds to the proton on the top part of the molecular structure with distances of 1.09 and 1.66 Å from heteroatoms) after being transferred mediates a strong O^–^–NH_3_^+^ H-bond between the two AA anions. The other panels instead show two proton migrations without the formation of an H-bond.

**Figure 11:**
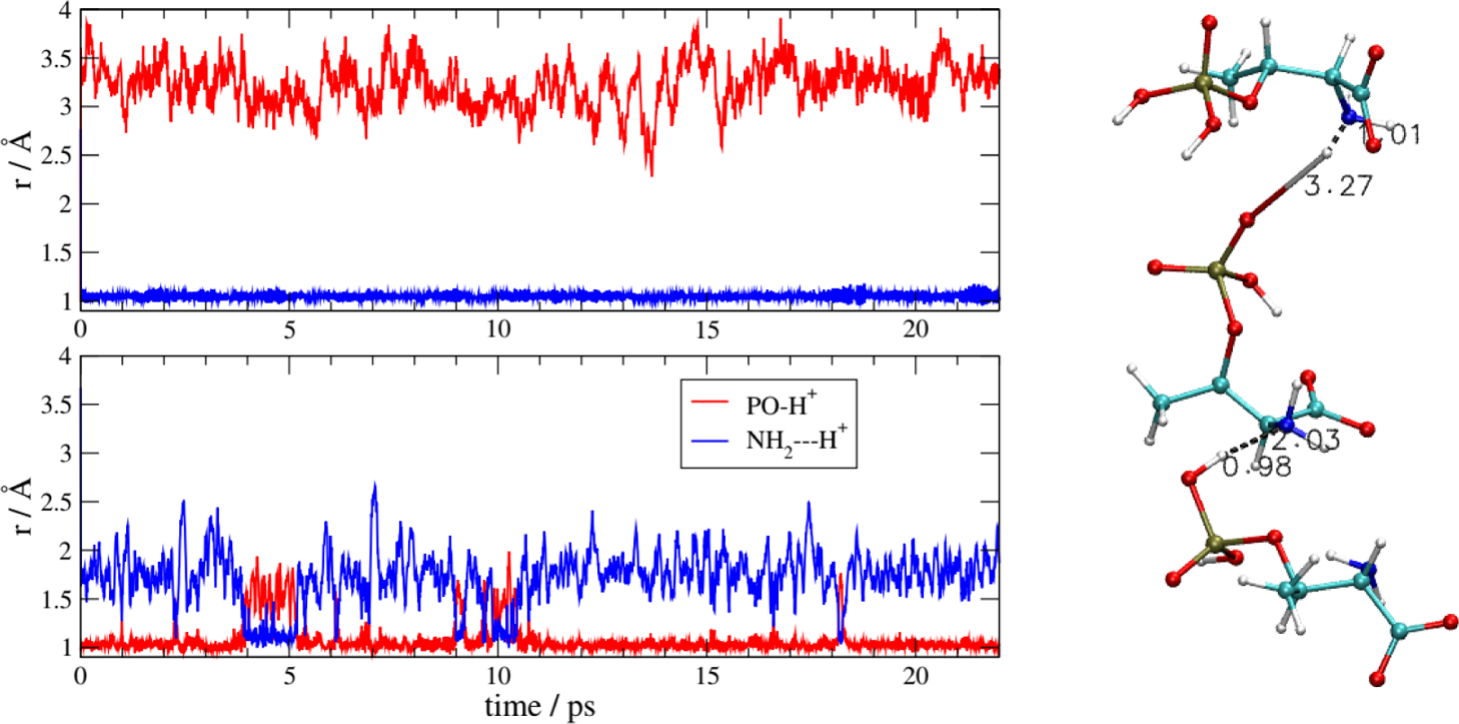
[Ch][Pth]. Left: proton–oxygen and proton–nitrogen distances as function of the simulation time. Right: relevant anionic cluster where the proton migration occurs as extracted from the final frame of the simulation.

The simulation for the [Ch][Pth] liquid reveals a very similar situation with the same proton migration mechanisms that we have observed in the Pse liquid. A peculiar situation worth noticing is depicted in Figure 11. With the usual notation of previous figures, we highlight the proton migration mechanisms in a cluster made by three AA anions. The interesting thing about this cluster is that, as we see in the lower panel, one of the phosphate-to-amino proton transfers is reversible and incomplete (lower panel). This behavior (completely unexpected, given the p*K*_a_’s at play) is very likely due to the above-mentioned tendency of the bulk phase to remain ionized.

## 5 Semi-Empirical Molecular Dynamics (DFTB): Toward Thermodynamics

The main problem with the ab initio computations presented above is that the simulated portions of the liquid are far from equilibrium and the simulation time is not long enough to reach a steady state for the various proton-transfer equilibria. Many of the events that we have seen taking place are dependent upon the initial state of the simulation cells. Unfortunately, at the present time, the calculation presented above represents the state-of-the-art data for AIMD. Such data have been collected using highly parallel computational resources and consuming nearly 4000 core/hours per day over a period of several months.

**Figure 12:**
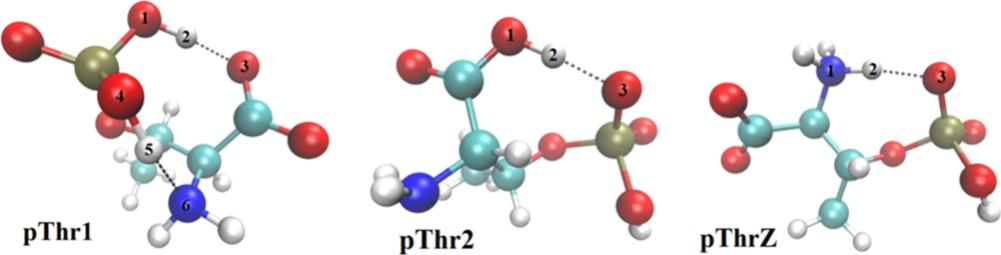
Minimum energy isomeric structures of the [Pth]^−^ anion as computed at B3LYP-D3/6-311+G(d,p) in PCM with ε = 35.7.

In order to unravel the thermodynamics of these systems, a simplification of the computational scheme is necessary. DFTB is a semi-empirical method that is way cheaper in terms of computational resources than DFT. As we have shown in Section S1, its accuracy is sufficient to provide an alternative viable way to simulate longer timescales. Given that the results collected for [Pse]^−^ and [Pth]^−^ point to a very similar structural and dynamic organization of the bulk phases, we have restricted the analysis with DFTB to the [Pth][Ch] liquid only. As we have shown above, the AA anion can appear in the liquid under three different tautomeric forms: an isomer has a deprotonated carboxylate (COO^–^, **pThr1**), another has a deprotonated phosphate (PO_4_H^–^, **pThr2**), and the last is a zwitterionic anion with both acids deprotonated and a protonated amino group (COO^–^, PO_4_H^–^, NH_3_^+^, **pThrZ**). The three tautomers of [Pth]^−^ are shown in Figure 12.

Given the prohibitively long times requested to reach an equilibration between these three possible states of the anionic moiety, we have performed 3 nonequilibrium simulations that differ in the initial conditions: the first one was set up with only **pThr1** anions, the second with **pThr2** anions, and the third with **pThrZ** ones. In this way, we have been able to sample the relative stability of the three isomers in the bulk phase and measure the likelihood of proton-transfer processes for each of them. The simulation with **pThr1** anions behaves in a similar manner to the ab initio ones described previously: proton transfers take place from the phosphate to carboxylates and amino groups in much the same way we have described before (we report some of these processes in Figures S1–S4 in the same fashion as we did for the AIMD ones above). The simulation with **pThr2** isomers behaves similarly: the proton is now initially on the carboxylate, and the phosphate group is negatively charged, but the interconversion between **pThr1** and **pThr2** is a fast and reversible process. This result is not unexpected given that the two isolated minimum geometries of the two isomers differ for less than 1 kcal/mol (see Table 1, last column).

In both the **pThr1** and **pThr2** simulations, we have noticed that the proton transfer to the amino group is much less reversible than the other migration pathways. In other words, when the proton has moved to an amino group, it tends to remain there and its mobility is greatly reduced. The proton transfer to an amino group leads to the formation of the zwitterionic anion **pThrZ,** which is a species, when taken in isolation, more stable than **pThr1** (COO^–^) and **pThr2** (PO_4_H^–^) (see Tables 1 and S1).

**Figure 13:**
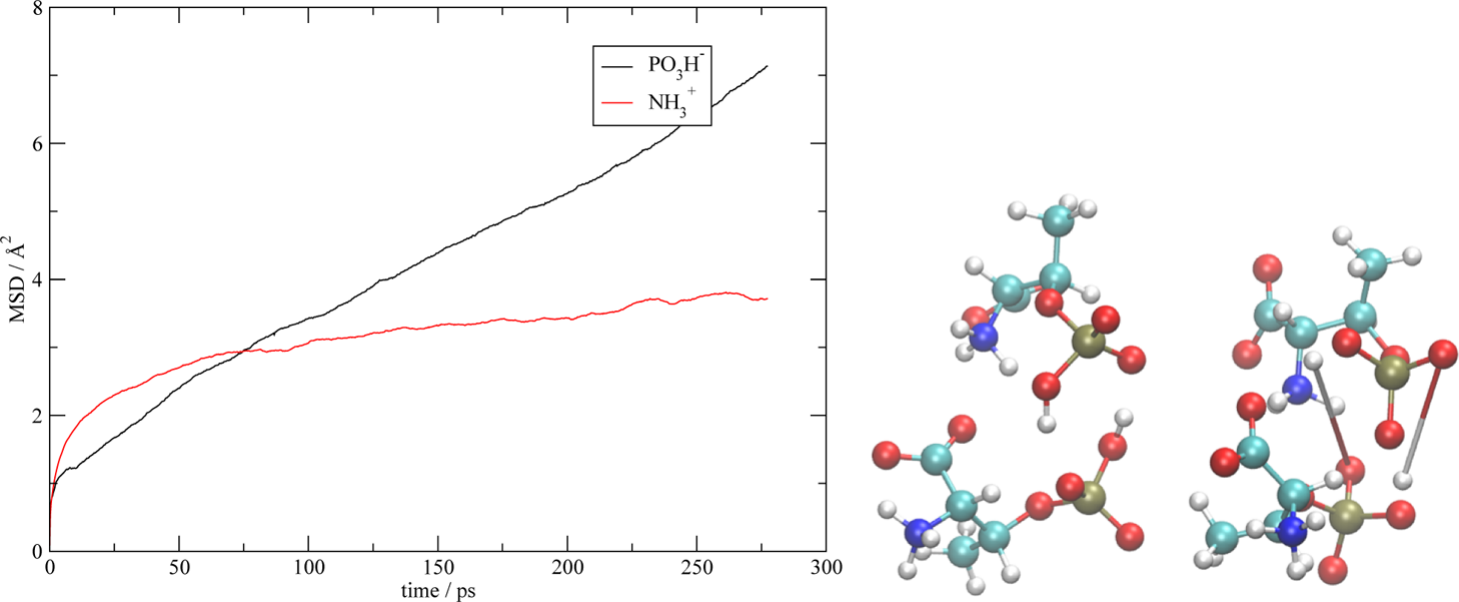
Left: mean square displacement of the PO_3_H^–^ and NH_3_^+^ protons in the pThrZ simulation. Right: snapshots of the anionic cluster where proton transfer occurs. The elongated distances in the right are drawn in order to easily trace the original positions of the two protons before the transfer.

It is for this reason that we have also performed a simulation in which all the anions are initially in their **pThrZ** form. What we have found is that all protons initially located on the amino group do not migrate within 200 ps of simulation time. This means that the thermodynamically stable form of the liquid is that made of **pThrZ** zwitterionic anions rather than the one that contains a mixture of **pThr1** and **pThr2** isomers. In turn, this also means that proton conductivity is actually quenched by the presence of the −NH_2_ basic groups, which act as scavengers of mobile protons. A form of residual conductivity, however, is still present because the phospho-AA have two protons on the PO_4_H_2_ group. Actually, we have found that, on longer timescales than those sampled by the ab initio simulations, the secondary proton on phosphate is able to migrate between the PO_4_H^–^ groups. The mechanism of proton migration is shown, with an example, in Figure 13 on the right: we have plotted the same two anions at different times along the simulation: initially (left structure), the two anions are clustered by H-bonds; during the simulation, a double exchange of the residual proton on the phosphate group takes place, resulting in the structure on the right. In order to further prove the existence of this residual proton mobility, in Figure 13 (on the left) we have also reported the mean square deviation of the proton positions of the phosphate along with those on the amino group. We see that those attached to the amino group (red line), after an initial displacement due to geometrical relaxation, reach a saturation regime, where their residual motion is simply due to the very limited drift of the anions in which they are contained. On the contrary, the protons initially attached to the negative phosphate show the typical linear trend of diffusive motion. Therefore, we conclude that, despite a complete protonation of the amino group, in these liquids, a mechanism able to sustain a limited conduction may still exist.

## 6 Conclusions

We have computed, by means of ab initio and semi-empirical MD, the structure of the bulk phase of two PILs made by [Ch]^+^ and phospho-AA anions. Within the limited time span accessible to our computations, we have also described the rather complicated dynamics of proton transfer that characterize such fluids. Two different types of simulations have been used: (i) those based on an ab initio computation of the forces (computationally very expensive) aimed at describing the short-time relaxation phenomena, and (ii) those based on a semi-empirical evaluation of the forces (computationally more efficient) that provided dynamics on longer timescales. The results we have obtained can be summarized as follows:

- The cohesive forces at play in these fluids are due to electrostatic and hydrogen bonds. The latter acts between anions and cations, but also between anions. Since the anions have more than one acceptor/donor functions, the ensuing network of hydrogen bonds is very complex.
- Hydrogen bonds between anions are strong enough to overcome the electrostatic repulsion (weakened by the dielectric screening and by charge delocalization) and cause the anions to cluster in small chains made by 3 or 4 ions.
- Within these chains, a complex dynamics of proton transfer takes place. The side chain of the AA is a phosphate, which is a good proton donor. Proton migration from this groups toward other groups is a fast and likely event in the fluid.
- Proton migration is a nearly reversible process when carboxylate and phosphate groups are involved, but it turns out to be irreversible when the acceptor is an amino group. In time, due to the amino group capturing protons from the acid groups, the fluid ultimately is made up of AA zwitterionic anions and [Ch]^+^. The presence of amino groups effectively quenches the proton transfers and reduces the overall conductivity of the fluid.
- A residual proton mobility survives even between zwitterionic anions because the secondary proton on the phosphate can still be exchanged between anions.

Overall, this study has elucidated the nanoscopic structure of a class of PILs. As we have already shown for other AA-based PILs, the presence of a series of tautomeric forms of the anions leads to complexity and to a charge distribution arrangement that is less obvious than one could initially assume based on an isolated molecules analysis. In particular, the emergence of such complex isomeric patterns depends on the presence of additional protic groups on the side chain. If these groups are good proton donors, the fate of each AA anions in the fluid will eventually be that of being transformed into a zwitterionic anion with two negative charges on acid groups and a positive one on the amino group. This conclusion represents an important novelty about the structure of this type of PILs. The unconventional charge distribution on the anionic moiety can have important consequences in their use as CO_2_ scavengers or as an electrochemical solvent.

## Supporting Information Available

Validation of the DFTB method against B3LYP calculations, relative energies in kcal/mol of the tautomeric forms of the two AA anions from DFTB and B3LYP, PA in kcal/mol of the various deprotonated states of the two AA from DFTB and B3LYP, DFTB semi-empirical simulations, X–H distances for various proton donor/acceptors as a function of time for various proton-transfer mechanisms and respective snapshots from the MD trajectories, and same data with an extended range of distances (PDF)
