# Supplementary material for: Ab Initio Molecular Dynamics Study of Phospho-Amino Acid-Based Ionic Liquids: Formation of Zwitterionic Anions in the Presence of Acidic Side Chains
Source: J Phys Chem B. 2020 Feb 10;124(10):1955–64. doi: 10.1021/acs.jpcb.9b09703 (PMC7997564; doi:10.1021/acs.jpcb.9b09703)
Supplement: Supplementary file 1 — jp9b09703_si_001.pdf [file jp9b09703_si_001.pdf]

An Ab-Initio Molecular Dynamics Study of Amino-acid Based Ionic Liquids: Formation of Zwitterionic Anions in the Presence of Acidic Side Chains

Henry Adenusi[a], Andrea Le Donne[a], Francesco Porcelli[a], Enrico Bodo[a *]

[a] *Chemistry Department, University of Rome "La Sapienza", Piazzale Aldo Moro 5, 00185, Rome Italy*

Supporting Information

S1 Validation of DFTB

In order to validate the semi-empirical method DFTB we have computed the relative energy of the possible tautomeric forms of the [Pth] AA anion using the well tested D3-B3LYP/6-311+G(d,p) method and compared the results with DFTB findings. The results are summarized in Table S1 where we report the energy differences between the various deprotonated states of the AA and the anionic structure, arbitrarily chosen as the reference one. The relative stability of the three tautomers of $[Pth]^-$, as assessed by B3LYP, is reproduced decently by DFTB with the ZW structure in the DFTB model being competitive in terms of energy with PO_4H^-. Overall, we find that the performance of the approximate DFTB model is good enough to describe the relative energies of the tautomeric forms of the anions.

	**B3LYP**		**DFTB**	
	vacuo	PCM	vacuo	PCM
COO^-	0	**0**	0	**0**
PO_4H^-	-2.7	**-0.5**	-2.2	**-1.3**
ZW	4.7	**-3.0**	2.3	**-0.9**

Table S1:Relative energies in kcal/mol of the tautomeric forms of the two AA anions. The tautomer labeled COO^- is a deprotonated AA that has lost the carboxylate proton, the one labeled PO_4H^- has lost the proton on the phosphate, the tautomer labeled ZW is an anionic zwitterion with the $COO^-/PO_4H^-/NH_3^+$ combination.

A further validation of the DFTB model against B3LYP is provided here by calculations of the gas-phase proton affinities (PA) of the basic group in the AA anionic structure. The results are shown in Table S2. The PAs have been computed as minus the energy difference of the following processes:

$$[AA]^- + H^+ \rightarrow [AA]H$$

* Corresponding Author. Email: enrico.bodo@uniroma1.it

where $[AA]^-$ is the aminoacid anion. In the DFTB framework, we have taken into account that the energy of the proton is not zero and we adopted the value of 151.04 kcal/mol for the proton energy.[1] The error of DFTB with respect with B3LYP-D3 in calculating the PA of the various AA anions is within 4%.

	**PA (kcal/mol)**	
	**B3LYP-D3**	**DFTB3**
[Pse]-		
$R—COO^- + H^+ \rightarrow R—COOH$	315.54	327.03
$R—PO_4H^- + H^+ \rightarrow R—PO_4H_2$	315.99	326.00
$R—NH_2 + H^+ \rightarrow R—NH_3^+$	216.35	210.21
[Pth]-		
$R—COO^- + H^+ \rightarrow R—COOH$	318.89	331.58
$R—PO_4H^- + H^+ \rightarrow R—PO_4H_2$	316.15	328.26
$R—NH_2 + H^+ \rightarrow R—NH_3^+$	221.85	214.42

Table S2: Proton affinities in kcal/mol of the various deprotonated states of the two AA.

S2 Results from the bulk simulations obtained using the DFTB method.

This section reports a set of data in order to describe the various proton transfer processes taking place during the simulations of the [Ch][Pth] ionic liquid as obtained by DFTB methods. The data are presented reporting the distance of the proton from the acceptor and donor groups during a proton exchange. In order to help following the dynamics, we also report a set of selected snapshots of the molecular structures involved in the proton transfer.

a) **pThr1** simulation. Example of a phosphate→carboxylate proton transfer.

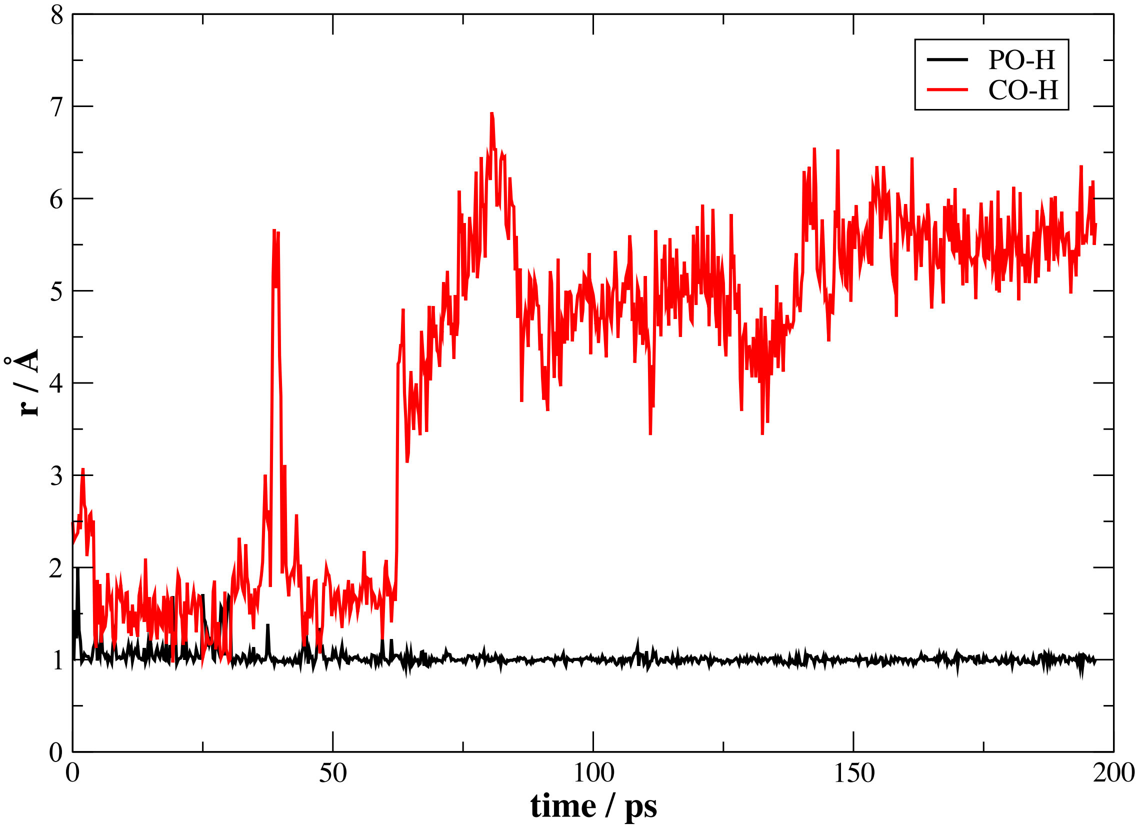

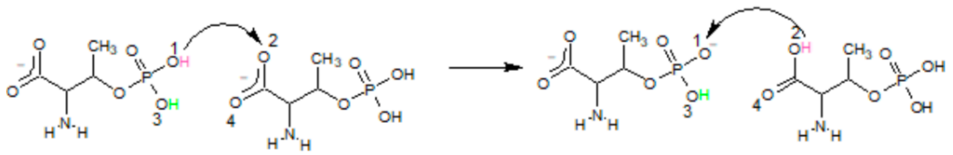

Figure S1: PO-H and CO-H distances as a function of time for a phospate→ carboxylate proton transfer in the ***pThr1*** *simulation taking place in a cluster of two anions (see Figure S2).*

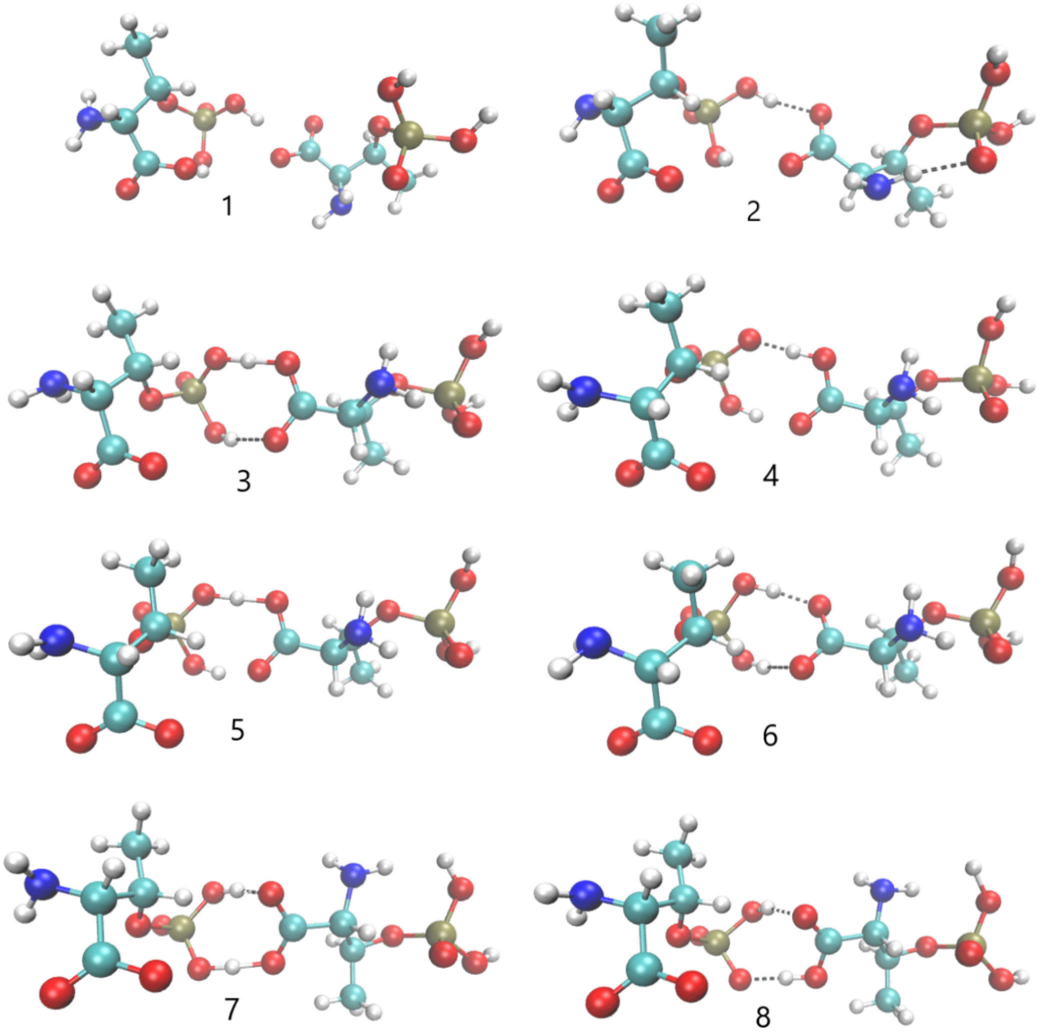

Figure S2: Actual snapshots of the cluster whose distances are reported in Figure S1.

b) **pThr1** simulation. Carboxylate→amino (top) and phosphate→amino (bottom) proton transfer with the formation of zwitterionic anions.

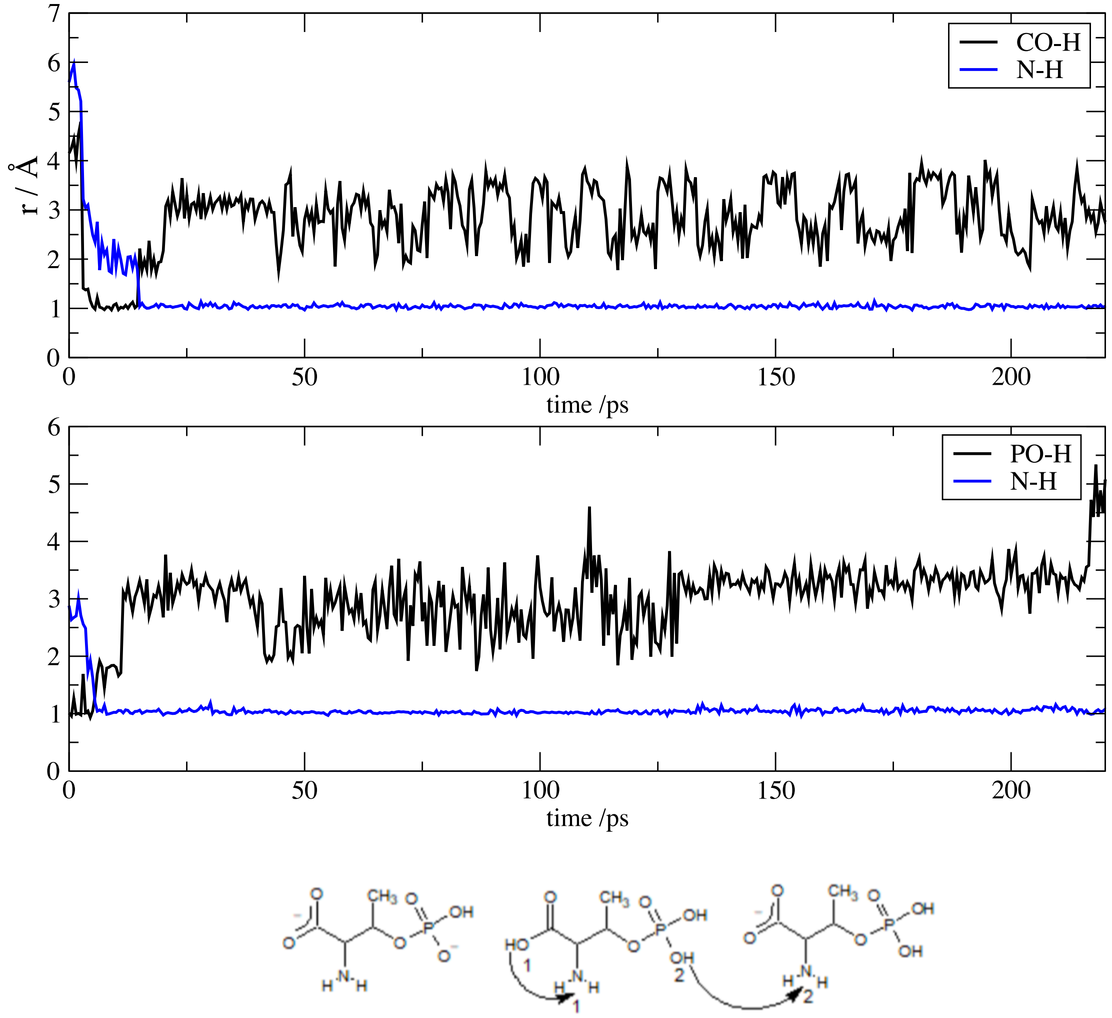

Figure S3: CO-H, PO-H and N-H distances as a function of time for two simoultaneous occurrences of proton transfer toward the amino group in the ***pThr1*** *simulation (see Figure S4, steps 7 and 8). The net effect is a tranfer from the phosphate to the amino group. Upper panel proton transfer labeled as 1; lower panel, proton transfer labeled as 2 in the scheme.*

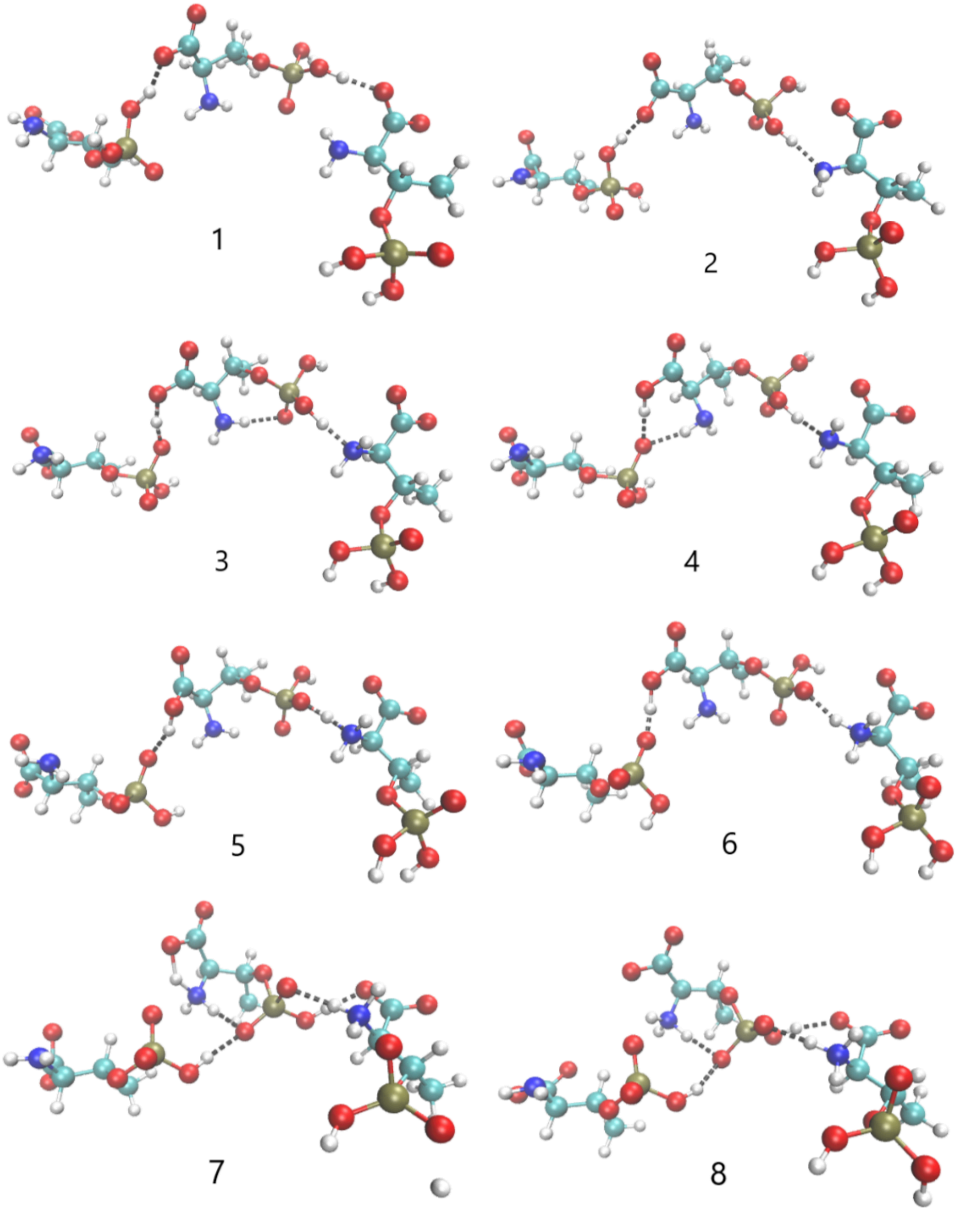

Figure S4: Actual snapshots of the cluster of three anions inside the bulk of the pThr1 simulation that give rise to the exchange of Figure S3.

c) **pThr2** simulation. Phosphate→amino proton transfer (formation of zwitterionic anions)

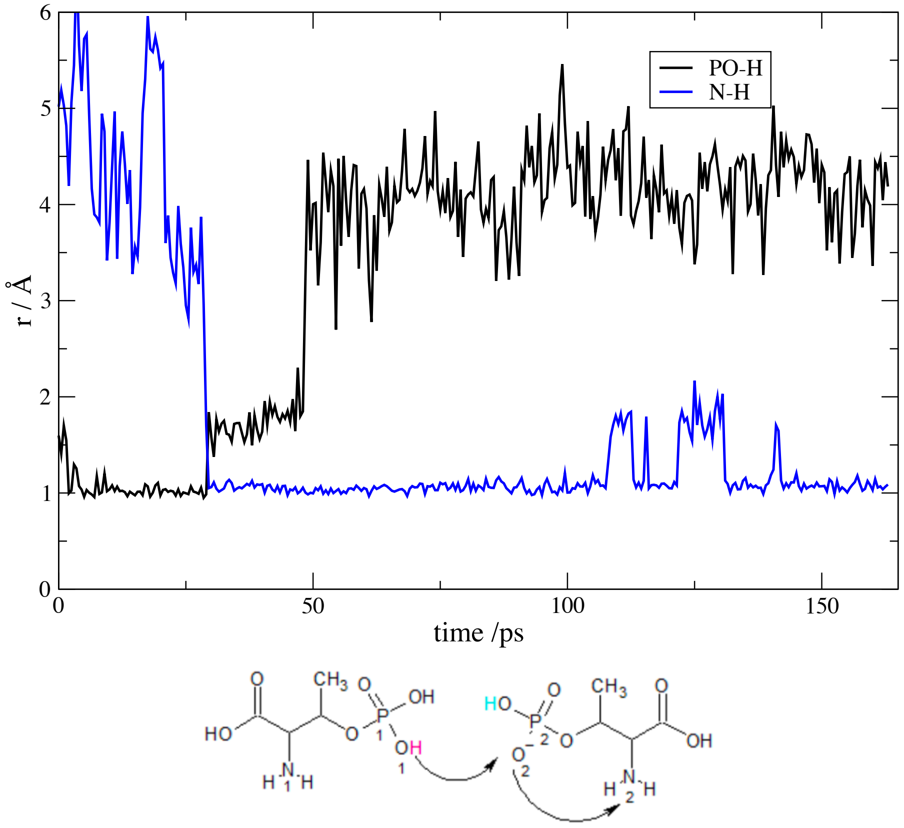

Figure S5: PO-H and N-H distances as a function of time for an intramolecular proton transfer toward the amino group in the pThr2 simulation taking place in a cluster made of three anions (see Figure S6, steps 1-7).

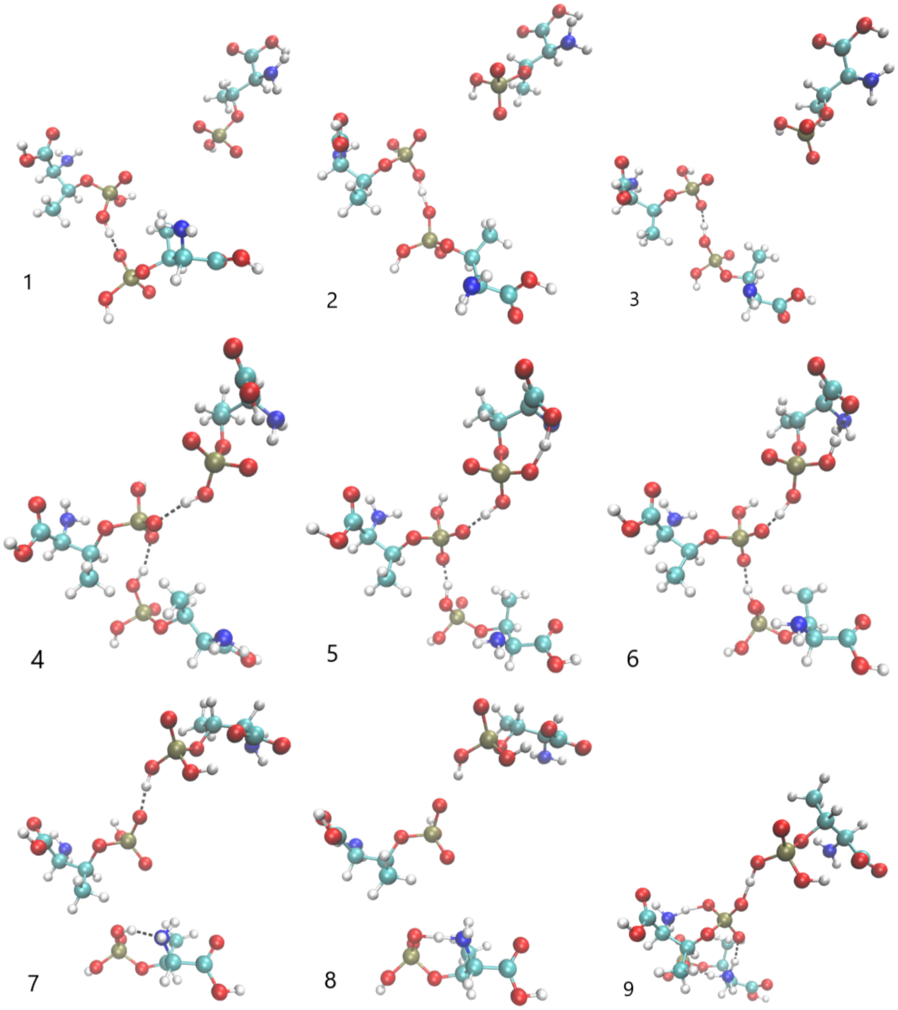

Figure S6: Snapshots of a cluster of three anions inside the bulk of the pThr2 simulation. Sequence of steps that in few ps lead to a phosphate→amino group proton transfer shown in Figure S5.

S3 Same data as in Figures 5, 6, 7 and 8 but on an enlarged distance scale

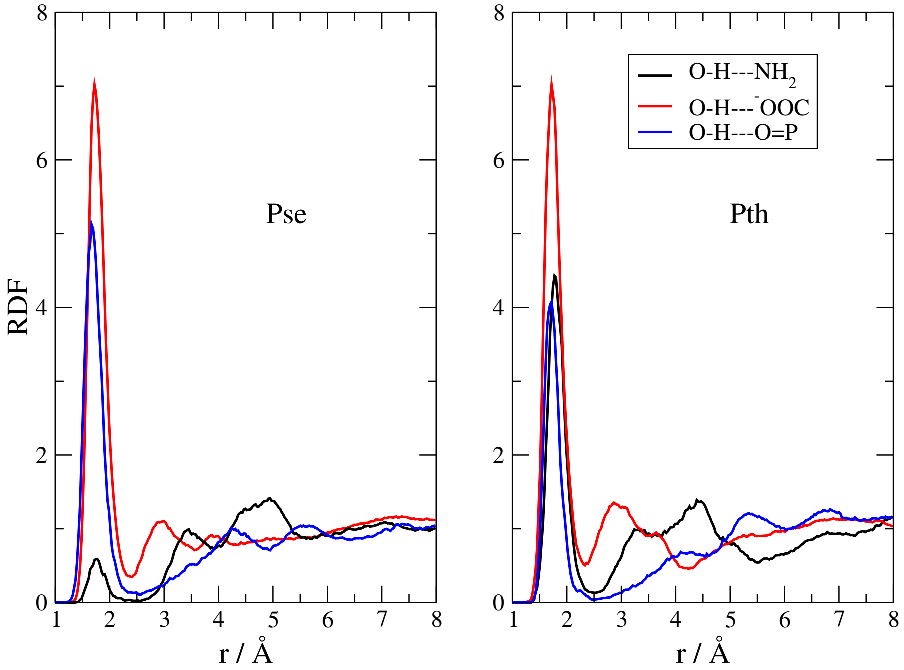

Figure S7: Same data as Figure 5, but in an extended range of distances.

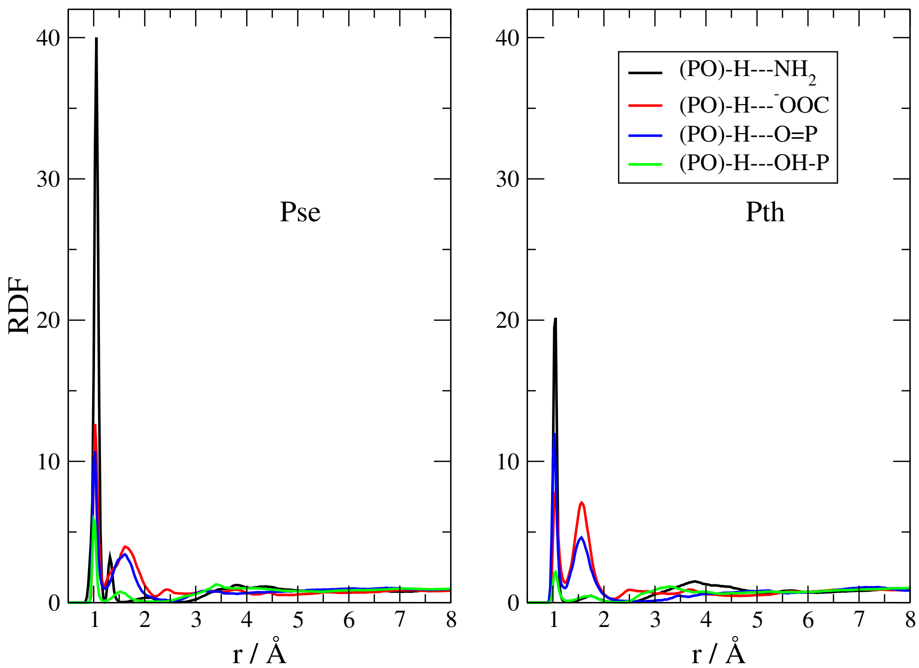

Figure S8: Same data as Figure 6, but in an extended range of distances.

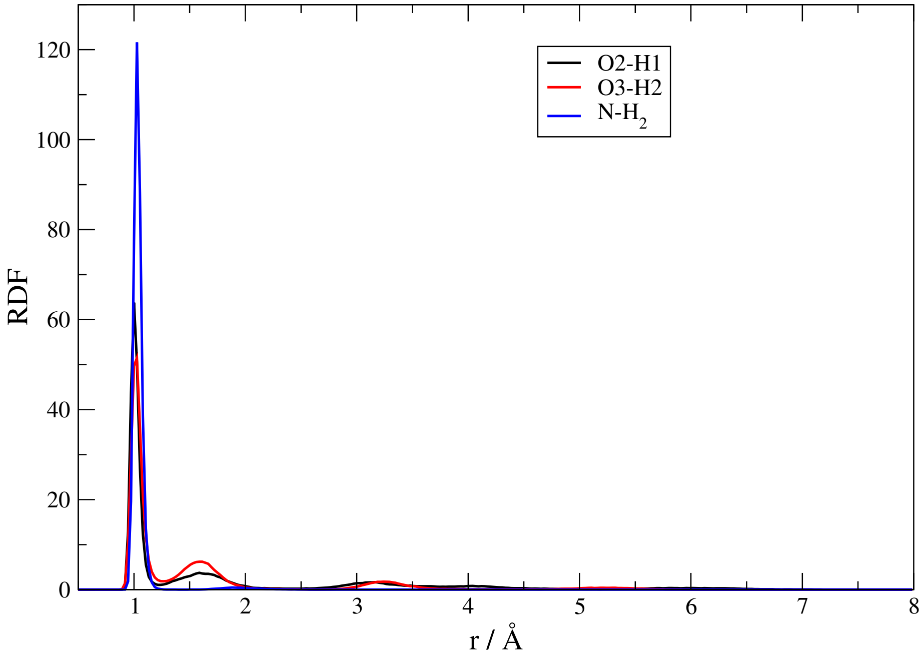

Figure S9: Same data as Figure 7, but in an extended range of distances.

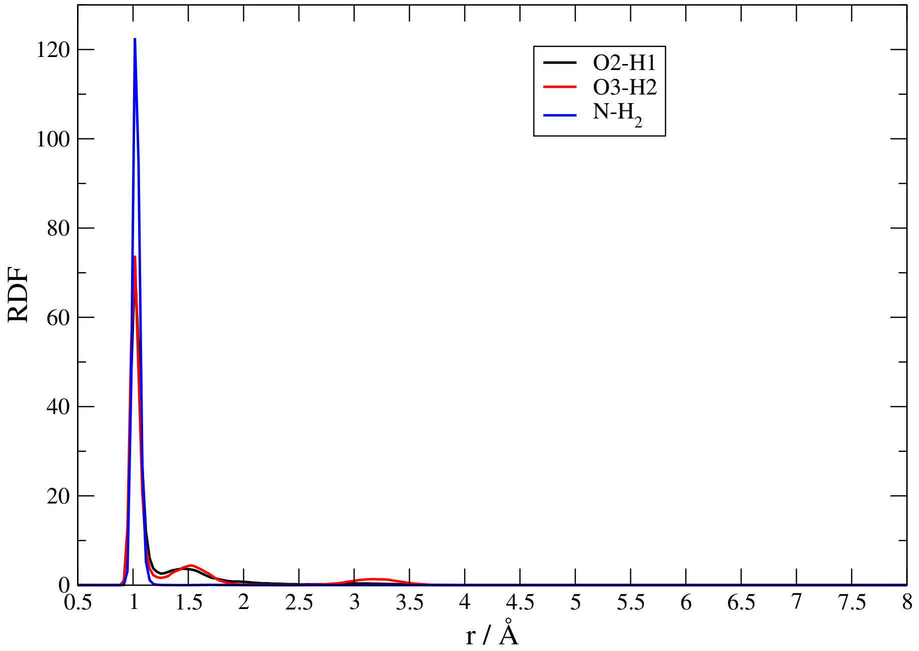

Figure S10: Same data as Figure 8, but in an extended range of distances.

---

[1] Addicoat, M. A.; Stefanovic, R.; Webber, G. B.; Atkin, R.; Page, A. J.; Assessment of the Density Functional Tight Binding Method for Protic Ionic Liquids, J. Chem. Theory Comput. **2014**, 10, 4633−4643
